# Enterovirus-associated changes in blood transcriptomic profiles of children with genetic susceptibility to type 1 diabetes

**DOI:** 10.1007/s00125-017-4460-7

**Published:** 2017-11-08

**Authors:** Niina Lietzen, Le T. T. An, Maria K. Jaakkola, Henna Kallionpää, Sami Oikarinen, Juha Mykkänen, Mikael Knip, Riitta Veijola, Jorma Ilonen, Jorma Toppari, Heikki Hyöty, Riitta Lahesmaa, Laura L. Elo

**Affiliations:** 1 Turku Centre for Biotechnology, University of Turku and Åbo Akademi University, Tykistökatu 6, FI-20520 Turku, Finland; 2 Department of Mathematics and Statistics, University of Turku, Turku, Finland; 3 Faculty of Medicine and Life Sciences, University of Tampere, Tampere, Finland; 4 Fimlab Laboratories, Pirkanmaa Hospital District, Tampere, Finland; 5 Department of Pediatrics, Turku University Hospital, Turku, Finland; 6 Research Centre of Applied and Preventive Cardiovascular Medicine, University of Turku, Turku, Finland; 7 Children’s Hospital, University of Helsinki and Helsinki University Hospital, Helsinki, Finland; 8 Research Programs Unit, Diabetes and Obesity, University of Helsinki, Helsinki, Finland; 9 Folkhälsan Research Center, Helsinki, Finland; 10 Tampere Center for Child Health Research, Tampere University Hospital, Tampere, Finland; 11 Department of Pediatrics, PEDEGO Research Unit, University of Oulu, Oulu, Finland; 12 Department of Children and Adolescents, Medical Research Center, Oulu University Hospital, Oulu, Finland; 13 Immunogenetics Laboratory, Institute of Biomedicine, University of Turku, Turku, Finland; 14 Department of Clinical Microbiology, Turku University Hospital, Turku, Finland; 15 Department of Physiology, Institute of Biomedicine, University of Turku, Turku, Finland

**Keywords:** Clinical immunology, Enterovirus, Human, Microarray, Prediction and prevention of type 1 diabetes

## Abstract

**Aims/hypothesis:**

Enterovirus infections have been associated with the development of type 1 diabetes in multiple studies, but little is known about enterovirus-induced responses in children at risk for developing type 1 diabetes. Our aim was to use genome-wide transcriptomics data to characterise enterovirus-associated changes in whole-blood samples from children with genetic susceptibility to type 1 diabetes.

**Methods:**

Longitudinal whole-blood samples (356 samples in total) collected from 28 pairs of children at increased risk for developing type 1 diabetes were screened for the presence of enterovirus RNA. Seven of these samples were detected as enterovirus-positive, each of them collected from a different child, and transcriptomics data from these children were analysed to understand the individual-level responses associated with enterovirus infections. Transcript clusters with peaking or dropping expression at the time of enterovirus positivity were selected as the enterovirus-associated signals.

**Results:**

Strong signs of activation of an interferon response were detected in four children at enterovirus positivity, while transcriptomic changes in the other three children indicated activation of adaptive immune responses. Additionally, a large proportion of the enterovirus-associated changes were specific to individuals. An enterovirus-induced signature was built using 339 genes peaking at enterovirus positivity in four of the children, and 77 of these genes were also upregulated in human peripheral blood mononuclear cells infected in vitro with different enteroviruses. These genes separated the four enterovirus-positive samples clearly from the remaining 352 blood samples analysed.

**Conclusions/interpretation:**

We have, for the first time, identified enterovirus-associated transcriptomic profiles in whole-blood samples from children with genetic susceptibility to type 1 diabetes. Our results provide a starting point for understanding the individual responses to enterovirus infections in blood and their potential connection to the development of type 1 diabetes.

**Data availability:**

The datasets analysed during the current study are included in this published article and its supplementary information files (www.btk.fi/research/computational-biomedicine/1234-2) or are available from the Gene Expression Omnibus (GEO) repository (accession GSE30211).

**Electronic supplementary material:**

The online version of this article (10.1007/s00125-017-4460-7) contains peer-reviewed but unedited supplementary material, which is available to authorised users.

## Introduction

Enteroviruses are among the most common viruses causing infections in humans. They are single-stranded RNA viruses that replicate typically in the intestine, but can occasionally spread also to blood and certain internal organs. Although enterovirus infections are mostly asymptomatic or cause only mild symptoms, they can also cause severe illnesses such as meningitis, myocarditis and hand-foot-and-mouth disease.

Several studies have associated viral infections, especially human enterovirus infections, with the development of type 1 diabetes [1–4]. Enteroviruses have a clear tropism to pancreatic beta cells [5], and low-grade enterovirus infection has been detected in pancreatic islets of living individuals with recently diagnosed type 1 diabetes [6]. Prospective studies have also found signs of enterovirus infections more commonly in children who later develop type 1 diabetes autoantibodies or clinical type 1 diabetes than in control children [1, 2, 7]. The presence of enteroviruses is not, however, thought to directly result in an increased risk of type 1 diabetes. The outcomes of infection likely depend on complex relationships between the host and the virus: for example, the genetic background and individual properties of the host [8–11], the timing of infections [12] and the type of enterovirus invading the host [13, 14]. Currently, only limited data are available regarding in vivo enterovirus responses in children at risk for developing type 1 diabetes, and most studies still rely on in vitro infection models. Therefore, better understanding of the individual-level responses to enterovirus infection is required to gain insights into the variable outcomes of these infections.

In this study, we performed, for the first time, genome-wide transcriptomic analysis of enterovirus-associated changes in children with genetic susceptibility to type 1 diabetes. We analysed microarray data from 44 longitudinally collected whole-blood samples [15] from seven children who were enterovirus-positive in one of the follow-up samples. Our aim was to understand the individual-level transcriptomic changes associated with enterovirus infections and to characterise the common features of enterovirus responses in young children.

## Methods

### Study participants and sample selection

The microarray data used in this study are part of the dataset published by Kallionpää et al [15] (GEO accession GSE30211) covering 356 PAXGene whole-blood RNA samples measured using the Affymetrix Human Genome U219 Array (Affymetrix, Santa Clara, CA, USA). The samples were collected from 28 pairs of children participating in the Finnish Type 1 Diabetes Prediction and Prevention (DIPP) study in Turku, Finland. All children in the DIPP study carry HLA-conferred genetic risk for type 1 diabetes, and they have been observed from birth at regular intervals [16]. All children had written parental consent and the Ethics Committee of Turku University Hospital had granted approval for the DIPP study. The study was carried out in accordance with the principles of the Declaration of Helsinki.

The presence of enterovirus RNA was studied using quantitative RT-PCR as described previously [17] from the same 356 RNA samples used for the microarray analyses. The RT-PCR was carried out in three parallel reactions. If all three reactions gave a positive result, the sample was classified as strongly enterovirus-positive; if only one of the reactions was positive, the sample was classified as weakly enterovirus-positive. In total, seven samples were detected as enterovirus-positive, each collected from a different child. Microarray data of all 44 samples from these enterovirus-positive children were selected for further analyses (electronic supplementary material [ESM] Table 1).

### Microarray data processing and clustering

The microarray data were pre-processed using the robust multiarray average (RMA) method implemented in the Bioconductor package affy version 1.44.0 (http://bioconductor.org/packages/release/bioc/html/affy.html), and log_2_-transformed. The Universal exPression Codes (UPC) method of the Bioconductor package SCAN.UPC version 2.12.1 (https://bioconductor.org/packages/release/bioc/html/SCAN.UPC.html) [18] was used to filter out probe sets with low expression (UPC < 0.5 in all the samples).

For each probe set, the RMA-normalised expression values were transformed into *z* scores based on their child-specific mean and standard deviation over the virus-negative samples. The *z* score profiles were clustered separately for each child using the k-means algorithm, with Pearson correlation and k = 10. For each child, the clusters with the highest and lowest centroid values at the time of enterovirus positivity were identified (referred to as peaking and dropping clusters, respectively).

### Functional data analysis

Functional classification of the data was performed using the Database for Annotation, Visualization and Integrated Discovery (DAVID; https://david.ncifcrf.gov/; accessed November to December 2016) [19] and Ingenuity Pathway Analysis (IPA; Qiagen Bioinformatics, Aarhus, Denmark, www.qiagenbioinformatics.com/products/ingenuity-pathway-analysis/; accessed November to December 2016) tools. Gene ontology classes with DAVID false-discovery rate (FDR) < 0.05 and IPA pathways with *p* value < 0.001 were considered significantly enriched. The Interferome 2.01 database (www.interferome.org; accessed November to December 2016) [20] was used to study the presence of human interferon-regulated genes.

### Microarray data from human PBMCs infected in vitro

The transcriptomics data from children at risk for type 1 diabetes were compared with microarray data (HumanHT-12 V3.0 BeadChip, Illumina, San Diego, CA, USA) from peripheral blood mononuclear cells (PBMCs) infected with enterovirus in vitro [14], including three replicate samples of PBMCs infected with ATCC strain of echovirus 9 or wild-type Coxsackie B1 virus strains CDC10802 and CDC10796 for 48 h, and uninfected control PBMCs (see Dataset 1 published on https://www.btk.fi/1234-2/). The data were pre-processed using the variance-stabilising normalisation of the Bioconductor lumi package version 2.18.0 (https://bioconductor.org/packages/release/bioc/html/lumi.html) [21]. The UPC method was used to filter out probes with low expression until reaching the number of probes equal to the dataset from the enterovirus-positive children. Differential expression was determined using the Bioconductor ROTS [22] package version 1.1.1 (https://bioconductor.org/packages/release/bioc/html/ROTS.html) and cut-off values FDR < 0.05 and fold change > 1.5. To enable comparison between the Illumina and Affymetrix platforms, the probes and probe sets were mapped to genes using IPA (Qiagen; accessed November to December 2016).

## Results

Enterovirus RNA was detected in seven of 356 whole-blood RNA samples, with five strongly enterovirus-positive and two weakly enterovirus-positive samples each taken from a different child.

**Fig. 1:**
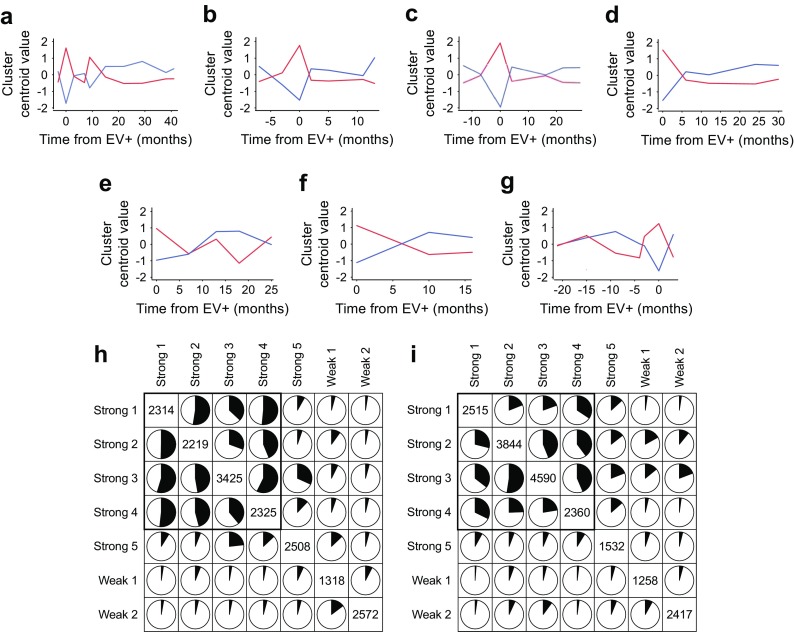
(**a**–**g**) Average expression profiles of clusters peaking or dropping at enterovirus positivity for the seven enterovirus-positive children: Strong 1 (**a**), Strong 2 (**b**), Strong 3 (**c**), Strong 4 (**d**), Strong 5 (**e**), Weak 1 (**f**) and Weak 2 (**g**). Red, peaking clusters; blue, dropping clusters. EV+, enterovirus-positive. (**h**, **i)** Overlapping probe sets between the peaking and dropping clusters. The black areas indicate the proportion of overlapping probe sets relative to the child/cluster noted at the top of the column. The boxes highlighted in the outlined frame show the peaking (**h**) and dropping (**i**) clusters of the four strongly enterovirus-positive children with the most similar enterovirus-associated changes. The total numbers of probe sets in each cluster are presented in the diagonal. The five children with strongly enterovirus-positive blood samples are denoted as Strong 1–Strong 5. The two children with weakly enterovirus-positive blood samples are denoted as Weak 1 and Weak 2

To characterise enterovirus-associated changes in whole-blood transcriptome, we studied longitudinal gene expression profiles of these enterovirus-positive children by dividing all probe sets child-specifically into ten clusters (ESM Fig. 1; see also Dataset 2 published on https://www.btk.fi/1234-2/). For each child, the clusters with the highest and lowest centroid value at the time of enterovirus positivity were selected as the enterovirus-associated signals (Fig. 1a–g).

For four strongly enterovirus-positive children, the overlaps between peaking and dropping clusters were higher (average overlaps of 46% and 37%, respectively) than those between the other children (average overlaps of 8%) (Fig. 1h, i). In total, 593 probe sets mapping to 339 distinct genes were detected in the peaking clusters of all four children. This set was defined as the enterovirus-induced signature. However, approximately 20% of the probe sets in each of these peaking clusters were child-specific, indicating the presence of individual differences in enterovirus responses. The other three children had lower overlaps with each other and with all other children.

**Fig. 2:**
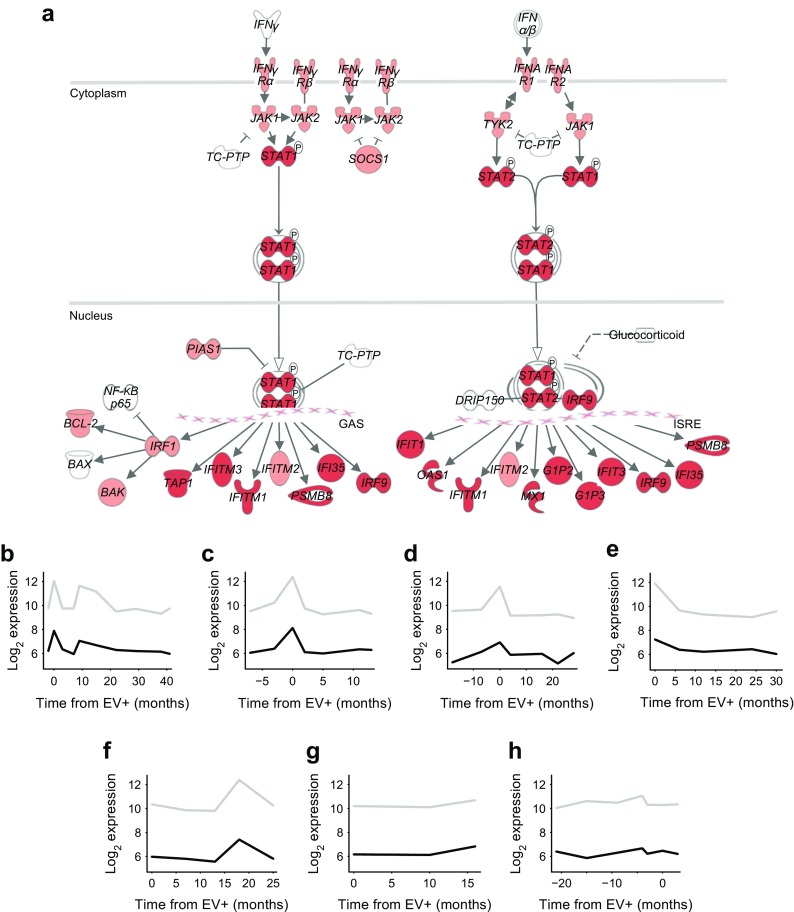
(**a**) Genes in peaking clusters mapping to the interferon signalling pathway based on the IPA tool. Red, genes present in at least four peaking clusters of the strongly enterovirus-positive children; pink, genes present in at least one peaking cluster. (**b**–**h**) Child-specific expression profiles of two genes of the interferon signalling pathway, *MX1* (grey) and *STAT2* (black). *IFNγ* is also known as *IFNG*; *IFNα/β* is also known as *IFNA1/B1*; *TC-PTP* is also known as *PTPN2*; *NF-κB p65* is also known as *RELA*; *BCL-2* is also known as *BCL2*; *BAK* is also known as *BAK1*; *DRIP150* is also known as *MED14*; *G1P2* is also known as *ISG15*; *G1P3* is also known as *IFI6*. EV+, enterovirus-positive; GAS, IFNG-activated sequence; ISRE, interferon-stimulated regulatory element. The five children with strongly enterovirus-positive blood samples are denoted as Strong 1–Strong 5 (**b**–**f**). The two children with weakly enterovirus-positive blood samples are denoted as Weak 1 and Weak 2 (**g**, **h**)

Genes involved in antiviral immune responses and especially interferon signalling were significantly enriched in both the peaking clusters of the four strongly enterovirus-positive children and the enterovirus-induced signature (Fig. 2a; see also Dataset 2 published on https://www.btk.fi/1234-2/). Also, child-specific expression profiles of two interferon signalling genes, *STAT2* and *MX1*, showed clear peaks in the four children at enterovirus positivity (Fig. 2b–h). Furthermore, 74% of the signature genes were regulated by interferons based on the Interferome database. The B cell receptor signalling pathway was among the most significantly enriched pathways in the peaking clusters of the other three children, potentially reflecting the activation of adaptive immune responses. Based on the dropping clusters, enterovirus infection was also associated with downregulation of genes involved in mRNA processing, transcription or translation in six of the seven children (see Dataset 3 published on https://www.btk.fi/1234-2/).

Enterovirus RNA is detectable in blood for only a few days during the acute phase of infection. To estimate the timing of infection relative to sample collection, we compared our peaking and dropping clusters with whole-blood transcriptional changes during acute and recovery phases after influenza virus infection, as reported by Zhai et al [23] (see Dataset 2 published on https://www.btk.fi/1234-2/). Of the 25 top genes upregulated during the acute phase of influenza infection [23], 23 overlapped our enterovirus-induced signature. Also, all seven natural killer (NK) cell activation signature genes associated with the acute phase of influenza infection [23] peaked in more than one of the enterovirus-positive children. Overlaps with the top up- and downregulated genes specific for the recovery phase after influenza virus infection [23] were low for all the children.

We also compared our results with enterovirus-induced responses in human PBMCs infected in vitro with three different enteroviruses [14]. The genes upregulated in the in vitro infections were enriched with those associated with the defence response to virus and the type I interferon signalling pathway, similarly to the genes in our enterovirus-induced signature. Of the genes upregulated by any of the enteroviruses, 70% were present in at least one of the peaking clusters. Overall, 77 genes present in the enterovirus-induced signature were upregulated in all three in vitro infections (ESM Fig. 2a). Of these genes, 73 were interferon-regulated based on the Interferome database. Although only approximately 50% of the genes downregulated in the in vitro infections were present in any of the dropping clusters of enterovirus-positive children, genes associated with translation were enriched in both datasets.

As enteroviruses are known to infect pancreatic islets [5] and have been found in pancreases of individuals with type 1 diabetes more often than in non-diabetic control groups [24, 25], we also compared our results with enterovirus-induced responses in human pancreatic islets infected in vitro with enteroviruses [10, 26]. Approximately half of the enterovirus-induced genes in human pancreatic islets [10, 26] were also present in our enterovirus-induced blood transcriptomic signature in children at risk for developing type 1 diabetes (ESM Fig. 2b), while the overlaps with the genes present in the peaking clusters of the other three type 1 diabetes risk children were low (ESM Fig. 2b). In total, there were 64 enterovirus-induced genes common to the in vitro infected pancreatic islets [10, 26] and our enterovirus-induced blood transcriptomic signature, all of which were associated with antiviral interferon responses, including *IFIH1*, *IRF7*, *MX1*, *STAT1* and *STAT2*.

**Fig. 3:**
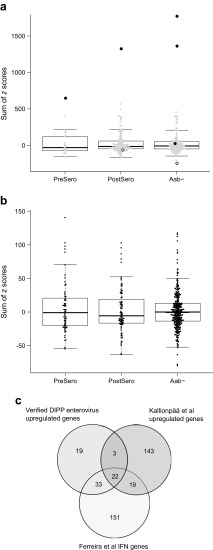
Expression of the 77 genes present in our enterovirus-induced signature and upregulated in all three in vitro enterovirus infections in microarray data published by: (**a**) Kallionpää et al [15]; and (**b**) Ferreira et al [27]. Sums of child-specific *z* scores over the 77 genes were calculated for each of the 356 whole blood samples by Kallionpää et al [15] (GEO: GSE30211) and the 454 PBMC samples by Ferreira et al [27] (Array Express: E-MTAB-1724), as described in Methods, using the published pre-processed datasets and sample information based on personal communications with Ferreira et al. All probes (**a**) or the highest-intensity exons mapping to genes (**b**) overlapping with the 77 genes were summed. (**a**) Black, strongly enterovirus-positive blood samples; white, weakly enterovirus-positive blood samples; grey, enterovirus-negative blood samples. (**a**, **b**) PreSero, samples collected from before seroconversion from children with autoantibody positivity or type 1 diabetes (in **a**, *n* = 22; in **b**, *n* = 65); PostSero, samples collected after seroconversion from children with autoantibody positivity or type 1 diabetes (in **a**, *n* = 169; **b** *n* = 84), Aab^−^, samples collected from autoantibody-negative children (in **a**, *n* = 165; in **b**, *n* = 305). (**c**) Venn diagram showing the overlaps between the 77 genes present in the enterovirus-induced signature and upregulated in all three in vitro enterovirus infections; genes upregulated before or after seroconversion in autoantibody-positive children based on the results by Kallionpää et al [15] and the 225 interferon-inducible genes detected by Ferreira et al [27]

Finally, we tested whether the 339 enterovirus-induced signature genes or the 77 genes also upregulated in all three in vitro infections of human PBMCs could differentiate between the enterovirus-positive and enterovirus-negative blood samples in the full transcriptomics dataset by Kallionpää et al [15]. The four enterovirus-positive samples with clear signs of interferon response activation were clearly separated from all other samples using either of the gene sets, while the other three enterovirus-positive samples were not separated from the enterovirus-negative samples (Fig. 3a, ESM Fig. 3a). To ensure that the observed separation was not due to the use of the same four enterovirus-positive children in defining the signature, a similar analysis was performed using the genes upregulated in the in vitro infected PBMCs by any of the three enteroviruses. Here also, the four strongly enterovirus-positive samples were clearly separated from the enterovirus-negative samples (ESM Fig. 3b).

Upregulation of interferon-regulated genes during enterovirus infection is one of the conspicuous features in this study, and activation of interferon signalling has been observed in the blood of children who have developed diabetes-related autoantibodies or clinical type 1 diabetes before the first detection of autoantibodies [15, 27]. However, the expression of the 339 enterovirus-induced signature genes (ESM Figs 3a, c and 4) or the 77 genes also upregulated with in vitro enterovirus infection (Fig. 3a, b) did not show marked differences between autoantibody-negative and autoantibody-positive children before or after seroconversion based on the longitudinal blood transcriptomics data from children at risk for type 1 diabetes reported by Kallionpää et al [15] or Ferreira et al [27]. Moreover, fewer than 50% of the genes upregulated in children who have developed diabetes-related autoantibodies or clinical type 1 diabetes in the two aforementioned studies [15, 27] overlapped with the enterovirus-induced signature (Fig. 3c, ESM Fig. 3c).

## Discussion

In the current study, we have identified enterovirus-associated transcriptomic profiles in whole-blood samples from seven children with genetic susceptibility to type 1 diabetes and characterised their individual responses to enterovirus infections.

Interferon response is a central part of the innate antiviral immune response, and several enterovirus strains induce a profound interferon response in human blood cells [14, 28]. We detected clear signs of interferon response activation in four strongly enterovirus-positive children. Enterovirus-associated changes in these children resembled previously reported differences occurring during the acute phase of virus infection [23], indicating that these samples were collected during the acute phase of infection characterised by high virus load. In two children with only weakly enterovirus-positive blood samples and one child with a strongly enterovirus-positive blood sample, no strong signs of interferon response were detected, but changes implying the activation of adaptive immune responses were observed. Enterovirus-associated downregulation of transcription, translation or mRNA processing-associated genes was observed in six children, although the individual probes and genes mapping to these categories varied between individuals. Upregulation of interferon response genes and downregulation of translation-associated genes were also detected in human PBMCs infected in vitro with three different enteroviruses. Finally, upregulation of genes associated with interferon responses was the common feature between enterovirus-induced blood transcriptomic changes in four children at risk for developing type 1 diabetes and in vitro enterovirus-infected human pancreatic islets [10, 26], creating a link between the virus–host interplay in blood and in the pancreas.

We built an enterovirus-induced signature covering 339 genes present in the peaking clusters of the four children with clear indications of interferon response activation, and a more selective signature of 77 genes additionally upregulated in human PBMCs infected in vitro with three different enteroviruses. Both signatures separated the four strongly enterovirus-positive samples from the other samples in the full microarray dataset published by Kallionpää et al [15].

The enterovirus-associated signature showed only moderate overlap with the upregulated genes in Kallionpää et al [15] and Ferreira et al [27], and could not differentiate between children who developed type 1 diabetes autoantibodies or clinical type 1 diabetes and autoantibody-negative children in those studies (ESM Fig. 3). Although activation of interferon signalling has been shown to precede the development of autoimmunity in children at risk for type 1 diabetes, our results indicate differences between enterovirus-associated and type 1 diabetes-associated interferon signals.

The four children with clear signs of interferon response activation included two persistently autoantibody-negative children, one child who later became positive for multiple type 1 diabetes autoantibodies and one autoantibody-positive child who later developed clinical type 1 diabetes. With the limited number of children available for the current study, and the significant amount of heterogeneity in enterovirus-associated changes between the children, it is not possible to draw conclusions regarding connections between enterovirus infections and type 1 diabetes.

There are several factors that can explain the observed heterogeneity in the enterovirus-associated responses. First, earlier in vitro studies have shown that the magnitude of interferon response induction in PBMCs varies significantly between different enteroviruses [14]. Second, the rapid kinetics of antiviral immune responses can be a source of significant heterogeneity when characterising enterovirus-associated blood transcriptomic changes in follow-up studies with long sampling intervals, although enterovirus RNA can be detected in blood for only a few days during the acute phase of infection. Third, host responses to acute infections caused by different viruses can be similar, and sometimes the divergences between viruses are better explained by the different magnitudes of the effect than by the actual genes responding to infection [23, 29]. Although our enterovirus-induced signature has a high overlap with in vitro enterovirus-induced changes in human PBMCs and pancreatic islets, we cannot conclude that these changes are uniquely observed after infection with enteroviruses. Finally, although enterovirus infections are often asymptomatic, clinical symptoms were reported for five of the seven children less than a week before the collection of the enterovirus-positive blood samples. Three children were suffering from fever around the time of enterovirus-positive sample collection, including one child also suffering from conjunctivitis, and common cold-like symptoms were reported for two children. Interestingly, the three children with fever around the time of enterovirus-positive sample collection were strongly enterovirus-positive based on quantitative RT-PCR and had clear signs of interferon response activation associated with the enterovirus-positive blood sample.

Despite the limitations of the current study, it provides a starting point for understanding the individual responses to enterovirus infections in vivo, and how these responses are reflected in the mRNA expression profiles in whole blood. Further longitudinal studies with larger cohorts, shorter sampling intervals and better knowledge of the actual virus strains infecting the individuals will provide deeper insights into the associations between enterovirus infections and type 1 diabetes.

## Electronic supplementary material

ESM (PDF 1196 kb)

## Abbreviations

DAVID: Database for AnnotationVisualization and Integrated Discovery
DIPP: Type 1 Diabetes Prediction and Prevention
FDR: False-discovery rate
IPA: Ingenuity Pathway Analysis
PBMC: Peripheral blood mononuclear cell
RMA: Robust multiarray average
UPC: Universal exPression Codes
