# Supplementary material for: Enterovirus-associated changes in blood transcriptomic profiles of children with genetic susceptibility to type 1 diabetes
Source: Diabetologia. 2017 Nov 8;61(2):381–8. doi: 10.1007/s00125-017-4460-7 (PMC6448961; doi:10.1007/s00125-017-4460-7)
Supplement: Supplementary file 1 — (PDF 1196 kb) [file 125_2017_4460_MOESM1_ESM.pdf]

ELECTRONIC SUPPLEMENTARY MATERIAL

Enterovirus-Associated Changes in Blood Transcriptomic Profiles of Children with Genetic Susceptibility to Type 1 Diabetes

Niina Lietzen[1,*], Le T. T. An[1,*], Maria K. Jaakkola[1, 2], Henna Kallionpää[1], Sami Oikarinen[3, 4], Juha Mykkänen[5, 6], Mikael Knip[7, 8, 9, 10], Riitta Veijola[11], Jorma Ilonen[12], Jorma Toppari[5, 13], Heikki Hyöty[3, 4], Riitta Lahesmaa[1†], Laura L. Elo[1,†]

[1] Turku Centre for Biotechnology, University of Turku and Åbo Akademi University, Turku, Finland

[2] Department of Mathematics and Statistics, University of Turku, Turku, Finland

[3] Faculty of Medicine and Life Sciences, University of Tampere, Tampere, Finland

[4] Fimlab Laboratories, Pirkanmaa hospital district, Tampere, Finland

[5] Department of Pediatrics, Turku University Hospital and University of Turku, Turku, Finland

[6] Research Centre of Applied and Preventive Cardiovascular Medicine, University of Turku, Turku, Finland

[7] Children's Hospital, University of Helsinki and Helsinki University Hospital, Helsinki, Finland

[8] Research Programs Unit, Diabetes and Obesity, University of Helsinki, Helsinki, Finland

[9] Folkhälsan Research Center, Helsinki, Finland

[10] Tampere Center for Child Health Research, Tampere University Hospital, Tampere, Finland

[11] Department of Pediatrics, PEDEGO Research Unit, Medical Research Center, Oulu University Hospital and University of Oulu, Oulu, Finland

[12] Immunogenetics Laboratory, University of Turku and Turku University Hospital, Turku, Finland

[13] Department of Physiology, Institute of Biomedicine, University of Turku, Turku, Finland

[*, †] Equal contribution

LIST OF ELECTRONIC SUPPLEMENTARY MATERIALS:

Electronic supplementary material Table 1.

Electronic supplementary material Figure 1.

Electronic supplementary material Figure 2.

Electronic supplementary material Figure 3.

Electronic supplementary material Figure 4.

Electronic supplementary material Table 1. Clinical data and sample collection information for the enterovirus-positive (EV+) children.

Child ID	Sampling ages (months)	Age at EV+ (months)	Gender	HLA risk class	T1D disease status	Age at T1D diagnosis (months)	ID in Kallionpää et al.*
**Strong 1**	13, 15, 18, 22, 24, 30, 37, 44, 53, 56	15	M	Moderate	AAB+	-	Case9
**Strong 2**	74, 78, 81, 83, 86, 92, 94	81	F	High	T1D	94	T1DCase4
**Strong 3**	50, 61, 68, 72, 84, 90, 96	68	F	Moderate	AAB-	-	Control7
**Strong 4**	37, 43, 49, 61, 67	37	M	Moderate	AAB-	-	T1DControl14
**Strong 5**	73, 80, 86, 91, 98	73	M	High	AAB-	-	T1DControl5B
**Weak 1**	14, 24, 30	14	M	Slightly increased	AAB-	-	T1DControl12
**Weak 2**	49, 55, 61, 66, 67, 70, 73	70	F	High	T1D	93	Case3

* Kallionpää *et al.*(2014) Diabetes. 63:2402-2414.

ESM Fig. 1

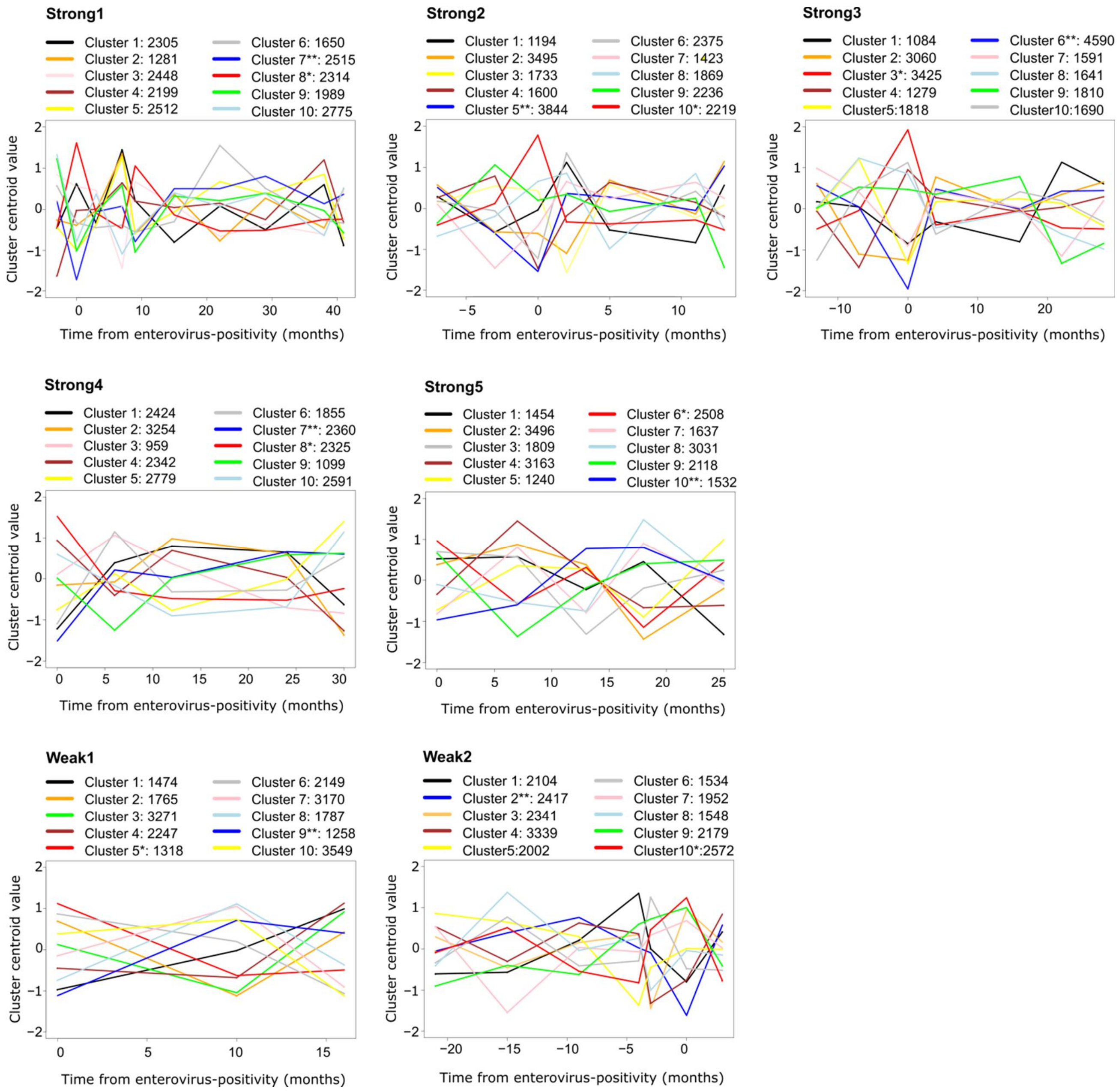

Electronic supplementary material Figure 1. Average expression profiles of the 10 probe clusters for each enterovirus-positive child. The number of probes in each cluster is given after the cluster name. *the peaking clusters for each child, **the dropping clusters for each child.

ESM Fig. 2

a

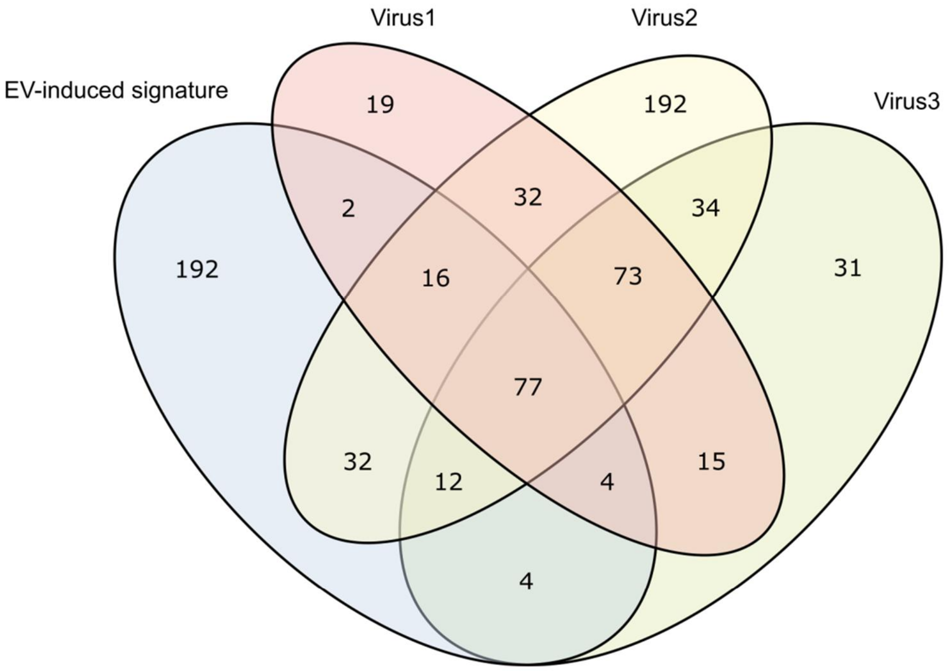

b

	Overlaps with genes in the peaking clusters of enterovirus-positive children			Overlaps with genes in the dropping clusters of enterovirus-positive children	
	PBMC up<br>Tot. 542 genes	Ylipaasto *et al.* up*<br>Tot. 436 genes	Domsgen *et al.* up**<br>Tot. 166 genes	PBMC down<br>Tot. 434 genes	Ylipaasto *et al.* down*<br>Tot. 510 genes
Strong1	256	179	106	56	75
Strong2	250	206	120	72	130
Strong3	233	175	97	84	129
Strong4	250	177	105	47	78
Strong5	51	32	6	44	38
Weak1	35	31	10	57	38
Weak2	42	33	6	47	68

* Ylipaasto *et al.* (2005) Diabetologia, 48: 1510-1522.
** Domsgen *et al.* (2016) Sci Rep, 6: 39378.

Electronic supplementary material Figure 2. a) Venn-diagram showing overlaps between the enterovirus-induced signature genes and the genes upregulated in the *in vitro* infected human PBMCs by Virus1 (Coxsackievirus B1 strain CDC10796), Virus2 (Coxsackievirus B1 strain CDC10802) and Virus3 (Echovirus 9). b) Numbers of overlapping genes between the peaking and the dropping clusters of the enterovirus-positive children and the differentially expressed genes in the *in vitro* enterovirus-infected PBMCs and pancreatic islets (Ylipaasto *et al.* [26], Domsgen *et al.* [10]).

ESM Fig. 3

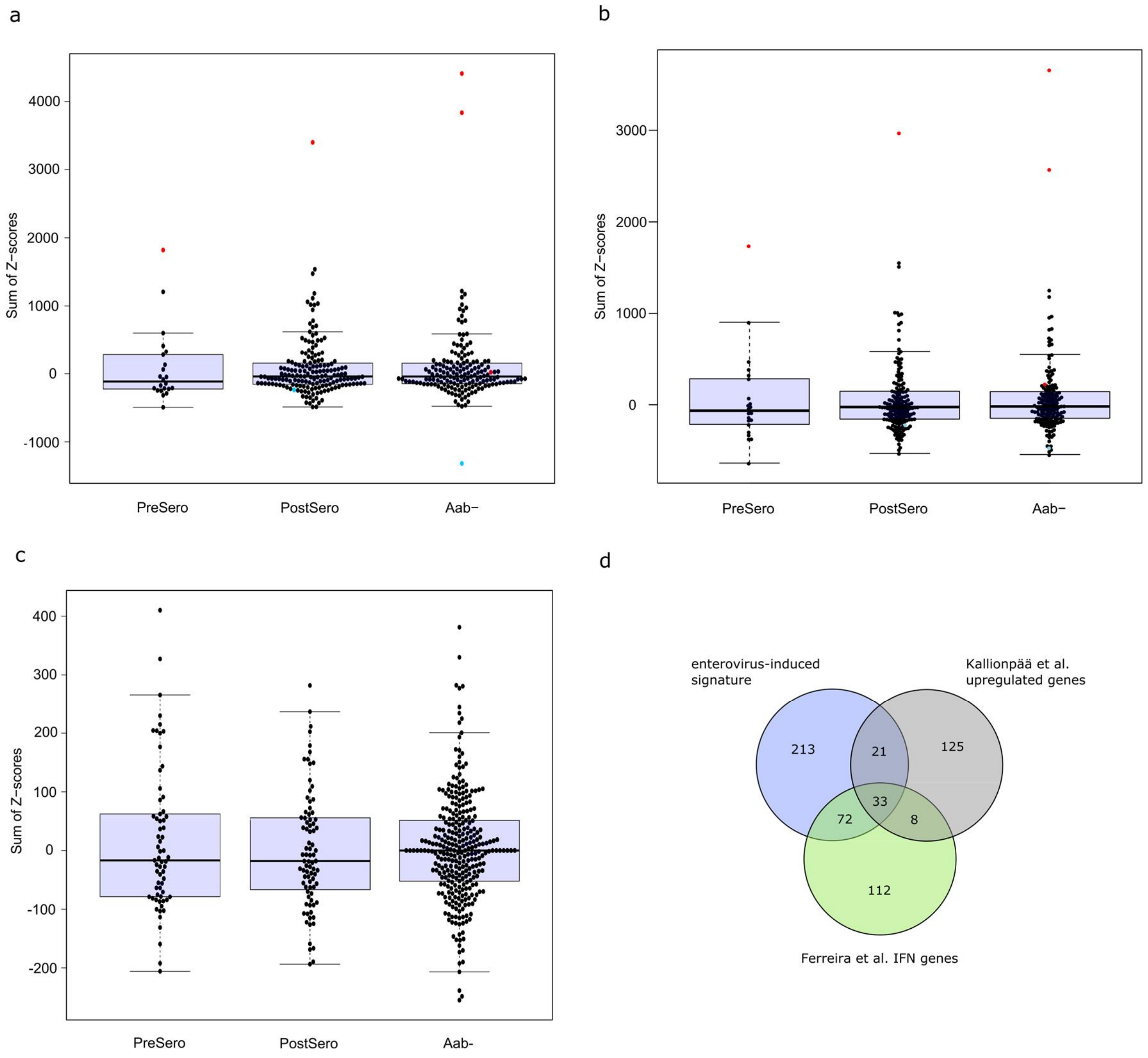

Electronic supplementary material Figure 3. Sums of child-specific Z-scores over the a) enterovirus-induced signature for each of the 356 whole blood samples by Kallionpää *et al.* [15] b) genes upregulated in the in vitro infected human PBMCs by any of the three enteroviruses for each of the 356 whole blood samples by Kallionpää *et al.* [15] (GEO: GSE30211) c) enterovirus-induced signature for each of the 454 PBMC samples by Ferreira *et al.* [27] (Array Express: E-MTAB-1724). Z-scores were calculated as described in the methods-section utilizing the published, preprocessed datasets and b) sample information based on personal communications with Ferreira *et al.* All probes (a, b) or the highest intensity exons mapping to genes (c) overlapping with the enterovirus-induced signature were summed. (a,b) red = strongly enterovirus-positive blood samples, blue = weakly enterovirus-positive blood samples, black = enterovirus-negative blood samples. (a-c) PreSero = samples collected from autoantibody-positive or T1D children before seroconversion (a-b: n = 22, c: n = 65), PostSero = samples collected from autoantibody-positive or T1D children after seroconversion (a-b: n = 169, c: n = 84), Aab- = samples collected from autoantibody-negative children (a-b: n = 165, c: n = 305). d) Venn diagram showing the overlaps between the enterovirus-induced signature and genes upregulated before or after seroconversion in autoantibody-positive children based on the analyses by Kallionpää *et al.* [15] and 225 interferon-inducible genes detected by Ferreira *et al.* [27].

ESM Fig. 4

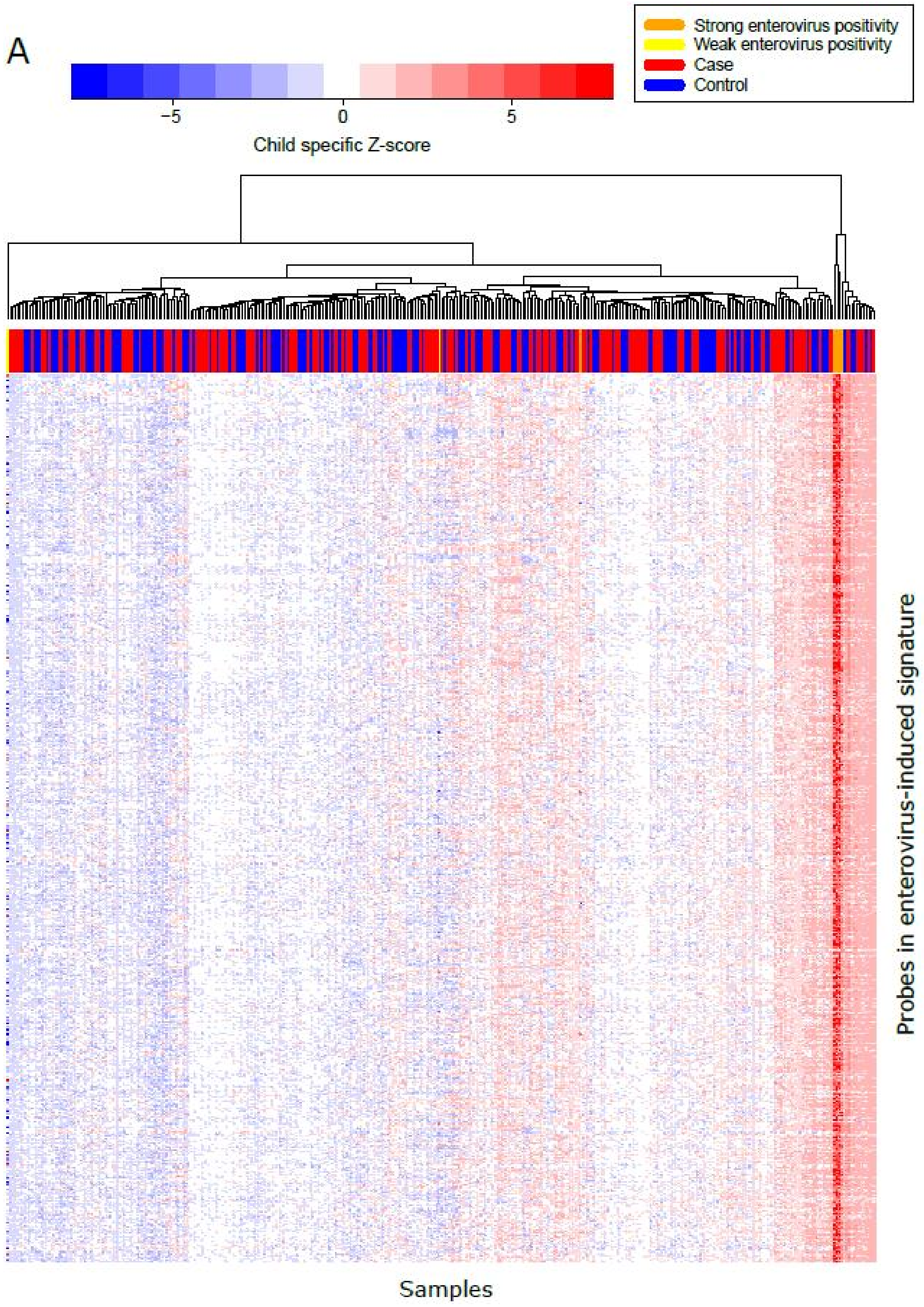
A
Strong enterovirus positivity
Weak enterovirus positivity
Case
Control
−5
0
5
Child specific Z-score
Probes in enterovirus-induced signature
Samples

B

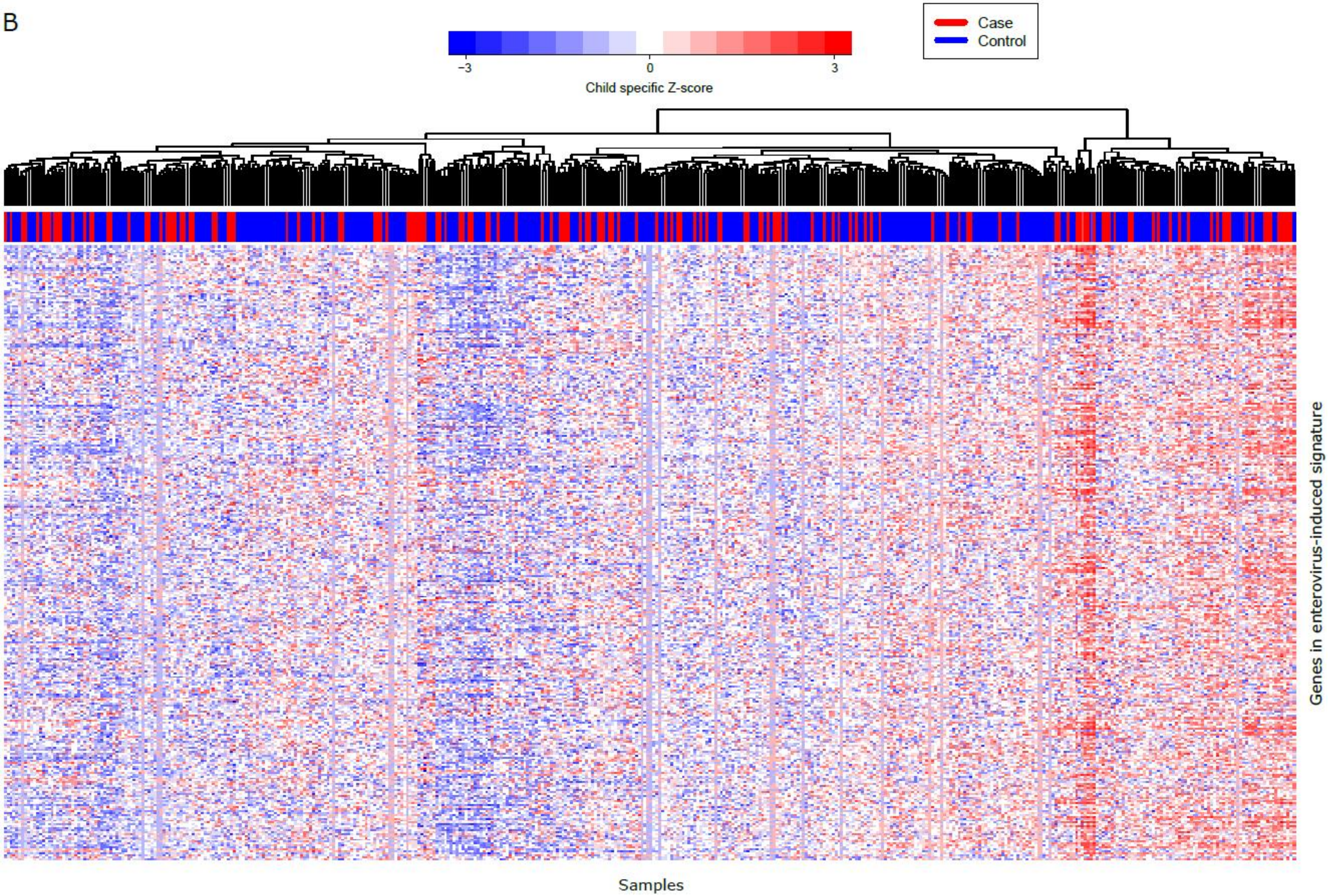

Electronic supplementary material Figure 4. Child specific Z-scores are illustrated as heatmaps where (a) probes or (b) genes in enterovirus-induced signatures are rows and all samples in (a) Kallionpää *et al.* [15] data or (b) Ferreira *et al.* [27] data are columns. Similarly to Figure 3, in Ferreira *et al.* [27] data (b), the exon with the highest intensity was selected to represent each gene in enterovirus-induced signature and child specific Z-scores were calculated as described in the methods-section. Case = autoantibody-positive children, control = autoantibody-negative children.
